# Intricate effects of primary motor neuronopathy on contractile proteins and metabolic muscle enzymes as revealed by label-free mass spectrometry

**DOI:** 10.1042/BSR20140029

**Published:** 2014-07-01

**Authors:** Ashling Holland, Thomas Schmitt-John, Paul Dowling, Paula Meleady, Michael Henry, Martin Clynes, Kay Ohlendieck

**Affiliations:** *Department of Biology, National University of Ireland, Maynooth, Co. Kildare, Ireland; †Department of Molecular Biology and Genetics, Aarhus University, Aarhus, Denmark; ‡National Institute for Cellular Biotechnology, Dublin City University, Dublin 9, Ireland

**Keywords:** amyotrophic lateral sclerosis, motor neuron disease, muscle proteomics, muscular atrophy, skeletal muscle proteome, wobbler mouse, ACN, acetonitrile, ALS, amyotrophic lateral sclerosis, GARP, Golgi-associated retrograde protein, LC, liquid chromatography, MLC2, myosin light-chain 2, MBP, myosin-binding protein, SERCA1, sarcoplasmic/endoplasmic reticulum Ca^2+^-ATPase 1, PI3K, phosphoinositide 3-kinase, SR, sarcoplasmic reticulum, TFA, trifluoroacetic acid, WR, wobbler, WT, wild-type

## Abstract

While the long-term physiological adaptation of the neuromuscular system to changed functional demands is usually reflected by unilateral skeletal muscle transitions, the progressive degeneration of distinct motor neuron populations is often associated with more complex changes in the abundance and/or isoform expression pattern of contractile proteins and metabolic enzymes. In order to evaluate these intricate effects of primary motor neuronopathy on the skeletal muscle proteome, label-free MS was employed to study global alterations in the WR (wobbler) mouse model of progressive neurodegeneration. In motor neuron disease, fibre-type specification and the metabolic weighting of bioenergetic pathways appear to be strongly influenced by both a differing degree of a subtype-specific vulnerability of neuromuscular synapses and compensatory mechanisms of fibre-type shifting. Proteomic profiling confirmed this pathobiochemical complexity of disease-induced changes and showed distinct alterations in 72 protein species, including a variety of fibre-type-specific isoforms of contractile proteins, metabolic enzymes, metabolite transporters and ion-regulatory proteins, as well as changes in molecular chaperones and various structural proteins. Increases in slow myosin light chains and the troponin complex and a decrease in fast MBP (myosin-binding protein) probably reflect the initial preferential loss of the fast type of neuromuscular synapses in motor neuron disease.

## Abstract 2

The systematic biochemical analysis of muscle from the wobbler mouse model of motor neuron disease suggests that the loss of neuromuscular synapses causes complex changes in the protein profile of contractile tissues, affecting especially the contractile apparatus and energy metabolism.

## INTRODUCTION

The continuum of mammalian skeletal muscles is characterized by distinct fibre-type-specific differences in physiological, biochemical and cellular properties [1–3] and this is reflected on the molecular level by highly complex and differential protein expression patterns [4–6]. Mature skeletal muscles exhibit a remarkable potential to adapt to changed functional demands, which is illustrated by swift transitions of muscle phenotypic profiles in response to altered neuromuscular activity levels [7–9]. While enhanced neuromuscular activity or chronic low-frequency electrostimulation causes a stepwise fast-to-slow transformation process in skeletal muscle, disuse-related muscular atrophy or complete denervation is typically associated with slow-to-fast transitions [10–14]. The detailed biochemical analysis of the sarcomeric myosin heavy chains has shown that hyper-excitability, denervation or muscular dystrophy cause very different changes in the isoform expression patterns of this essential contractile protein in skeletal muscles [15]. These biochemical consequences of fibre-type shifting are also evident by proteome-wide alterations in the abundance and isoform expression pattern of contractile proteins and metabolic enzymes, as outlined in several recent reviews on MS-based skeletal muscle proteomics [16–18]. The pathophysiology of preferential alterations in fibre innervation, such as observed during paralysis in motor neuron disease, appears to be associated with less unilateral changes in muscle fibres [19] and the denervation–reinnervation patterns in neurogenic atrophy are also associated with the presence of small fibres and the expression of immature isoforms of myosin heavy chains [20]. This complexity of molecular changes therefore requires systematic approaches for the comprehensive analysis of progressive neurodegeneration and its down-stream effects on contractile fibres within the affected motor units [21].

Motor neuron diseases are a heterogeneous group of neurodegenerative syndromes, such as hereditary spastic paralysis, primary lateral sclerosis, spastic paraplegia, spinobulbar muscular atrophy and ALS (amyotrophic lateral sclerosis). A highly progressive adult-onset loss of lower and upper motor neurons resulting in paralysis is characteristic of ALS and can be of genetic origin or be sporadic in nature [22–24]. Muscular atrophy leads to limb and bulbar muscle weakness and respiratory insufficiency. Various genes were shown to be involved in inherited forms of ALS, including *SOD1* (*superoxide dismutase 1*), *TARDBP*, *FUS, VCP*, *OPTN*, *ALS2*, *SETX*, *C9ORF72*, *PFN1*, *VAPB*, *UBQLN2* and *ANG* [25–28]. Genomic, transcriptomic, proteomic and metabolomic screening programmes have been initiated to determine global changes in ALS specimens and animal models of motor neuron disease [29,30] and to establish novel biomarkers for diagnostic, prognostic and therapeutic applications [31,32]. Proteomic profiling of the effects of progressive neurodegeneration has included cerebrospinal fluid, cervical spinal cord specimens, lumbar spinal cord preparations and skeletal muscles [33–40].

Rodent models of motor neuron disease play a crucial role in ALS research, as recently reviewed by McGoldrick et al. [41], including the WR (wobbler) mouse (genotype *wr*/*wr*, phenotype WR) [42,43]. The WR mouse exhibits progressive neurodegeneration and neuroinflammation [44–46] and is frequently used as an ALS model for testing novel pharmacological approaches [47–49], although no human neurodegenerative disease has yet been identified that exhibits the same genetic abnormality [50]. The primary alteration in the WR genotype has been identified as a missense mutation in the ubiquitously expressed gene *Vps54* that leads to a leucine-to-glutamine replacement (L967Q) near the C-terminus of the 977 amino acid polypeptide chain of VPS54 [51]. The vesicular protein sorting factor VPS54 represents an essential component of the hetero-trimeric GARP (Golgi-associated retrograde protein) complex and the hydrophobic-to-hydrophilic amino acid exchange in its primary sequence causes destabilization of its tertiary protein structure leading to a reduction in the concentration levels of VPS54 [52].

In analogy to the previous gel-based survey of the WR and SOD mouse models of ALS, using fluorescence 2D difference in-gel electrophoresis [39,40], we have used here a complementary gel-free method, i.e. label-free MS, to determine secondary changes in WR skeletal muscle tissue due to abnormal expression of the GARP component VPS54. The proteomic analysis verified the complexity of neurodegeneration-related changes in skeletal muscle from the WR mouse model of ALS and identified novel changes in proteins involved in fibre contraction, energy metabolism, metabolite transportation, ion handling, structural integrity and the cellular stress response.

## MATERIALS AND METHODS

### Materials

Materials and analytical-grade chemicals used for the mass spectrometric analysis of WR versus WT (wild-type) skeletal muscle tissues were obtained from Amersham Biosciences/GE Healthcare and BioRad Laboratories. The proteolytic enzymes Lys-C and trypsin were purchased from Promega. Protease inhibitors and chemiluminescence substrate were from Roche Diagnostics. Whatman nitrocellulose transfer membranes were obtained from Invitrogen. Primary antibodies were purchased from Abcam [ab55559 to MBP (myosin-binding protein) C; ab97708 to troponin TnT; ab48003 to MLC2 (myosin light-chain 2); ab8592 to desmin; ab41803 to annexin; ab26300 to lamin; ab6588 to collagen; ab13496 to αB-crystallin; ab85366 to carbonic anhydrase CA3; ab43176 to ATP-synthase; ab36329 to isocitrate dehydrogenase; and ab28172 to prohibitin] and Sigma Chemical Company (L-9393 to laminin). Secondary peroxidase-conjugated secondary antibodies were obtained from Chemicon International. All other chemicals were analytical grade and purchased from Sigma Chemical Company.

### WR mouse model of motor neuron disease

The origin of the WR stock and breeding of mice at Aarhus University has been previously described [53]. In this comparative study, 9-week-old WR mice and age-matched normal C57 mice were used. Animals were bred in accordance with Danish law on the protection of laboratory animals and approved by the local authorities. Mice were sacrificed by cervical dislocation and all biochemical and proteomic studies were carried out with *post-mortem* tissue samples.

### Preparation of tissue extracts from WR and WT muscle

Muscles from the hind limb of WR and WT mice were freshly dissected, quick-frozen in liquid nitrogen, transported on dry ice and then stored at −80°C prior to usage. Equal amounts of tissue (100 mg wet weight) were employed for the preparation of muscle extracts. Skeletal muscle tissue was homogenized using a hand-held IKA T10 Basic Homogenizer (IKA-Labortechnik). Crude extracts were then incubated for 2.5 h at room temperature with agitation using a Thermomixer from Eppendorf. Samples were centrifuged at 4°C for 20 min at 14 000 ***g*** and the urea-soluble protein containing-middle layer was retained for further analysis [54]. For comparative proteomic profiling, skeletal muscle homogenates from WR mice (n=4) and WT mice (n=4) were pre-treated with the Ready Prep 2D clean up kit from BioRad Laboratories. The resulting protein pellets were resuspended in label-free solubilization buffer, consisting of 10 mM Tris, pH 8.0, 6 M urea and 2 M thiourea in LC (liquid chromatography)–MS grade water [55]. Following vortexing, sonication and centrifugation, the protein concentration of the WR and WT suspensions was determined. Volumes of protein suspensions were equalized using label-free solubilization buffer and then reduced for 30 min with 10 mM DTT (dithiothreitol) and alkylated for 20 min in the dark with 25 mM iodoacetamide in 50 mM ammonium bicarbonate. The proteolytic digestion of proteins was carried out in two steps. First, digestion was performed with sequencing grade Lys-C at a ratio of 1:100 (protease/protein) for 4 h at 37°C, followed by a dilution with four times the initial sample volume in 50 mM ammonium bicarbonate. Secondly, further digestion was based on incubation with sequencing grade trypsin at a ratio of 1:25 (protease/protein) overnight at 37°C. The protease-treated muscle protein suspensions were diluted 3:1 (v/v) with 2% (v/v) TFA (trifluoroacetic acid) in 20% (v/v) acetonitrile. To ensure an even suspension of peptide populations from WR versus WT muscle, the samples were briefly vortexed and sonicated [55].

### Label-free LC–MS/MS analysis

The nano LC–MS/MS analysis of WR versus WT samples was carried out with the help of an Ultimate 3000 nanoLC system (Dionex) coupled to an LTQ Orbitrap XL mass spectrometrer (Thermo Fisher Scientific) in the Proteomics Facility of the National Institute for Cellular Biotechnology, Dublin City University. The optimized methodology has been previously described in detail [56,57]. Peptide mixtures (5 μl volume) were loaded onto a C18 trap column (C18 PepMap, 300 μm id×5 mm, 5 μm particle size, 100 Å pore size; Dionex). Desalting was achieved at a flow rate of 25 μl/min in 0.1% TFA for 10 min. The trap column was switched on-line with an analytical PepMap C18 column (75 μm id×500 mm, 3 μm particle and 100 Å pore size; Dionex). Peptides generated from muscle proteins were eluted with the following binary gradients: solvent A [2% (v/v) ACN (acetonitrile) and 0.1% (v/v) formic acid in LC–MS grade water] and 0–25% solvent B (80% ACN and 0.08% (v/v) formic acid in LC–MS grade water) for 240 min and 25–50% solvent B for a further 60 min. The column flow rate was set to 350 nl/min [56]. Data were acquired with Xcalibur software, version 2.0.7 (Thermo Fisher Scientific). The MS apparatus was operated in data-dependent mode and externally calibrated. Survey MS scans were acquired in the Orbitrap in the 300–2000 *m/z* range with the resolution set to a value of 30 000 at *m/z* 400 and lock mass set to 445.120025 u. CID (collision-induced dissociation) fragmentation was carried out in the linear ion trap with the three most intense ions per scan [56]. Within 60 s, a dynamic exclusion window was applied. A normalized collision energy of 35%, an isolation window of 3 *m/z* and one microscan were used to collect suitable tandem mass spectra [57].

### Quantitative profiling by label-free LC–MS/MS analysis

Processing of the raw data generated from LC–MS/MS analysis was carried out with Progenesis label-free LC–MS software (version 3.1; Non-Linear Dynamics). Data alignment was based on the LC retention time of each sample, allowing for any drift in retention time given and adjusted retention time for all runs in the analysis [56]. A reference run was established with the sample run that yielded most features (i.e. peptide ions). The retention times of all of the other runs were aligned to this reference run and peak intensities were then normalized. Prior to exporting the MS/MS output files to MASCOT (www.matrixscience.com) for protein identification, a number of criteria were employed to filter the data including: (i) peptide features with ANOVA<0.05 between experimental groups; (ii) mass peaks (features) with charge states from +2, +3; and (iii) greater than one isotope per peptide [55]. A MASCOT generic file was generated from all exported MS/MS spectra from Progenesis software. The MASCOT generic file was used for peptide identification with MASCOT (version 2.2) and searched against the UniProtKB-SwissProt database (downloaded in January 2013) with 16 638 proteins (taxonomy: *Mus musculus*). The following search parameters were used for protein identification: (i) MS/MS mass tolerance set at 0.5 kDa; (ii) peptide mass tolerance set to 20 ppm; (iii) carbamidomethylation set as a fixed modification; (iv) up to two missed cleavages were allowed; and (v) methionine oxidation set as a variable modification. For further consideration and reimportation back into Progenesis LC–MS software for further analysis, only peptides with ion scores of 40 and above were chosen [57]. Importantly, the following criteria were applied to assign a skeletal muscle-associated protein as properly identified: (i) an ANOVA score between experimental groups of ≤0.05; (ii) proteins with ≥2 peptides matched; and (iii) a MASCOT score >40 [55,56]. Standard bioinformatics software was used to identify the clustering of molecular functions of the MS-identified muscle proteins with a changed abundance in WR mouse. These analyses were performed with the PANTHER (http://pantherdb.org; version 8.1) comprehensive database of protein families for the cataloguing of molecular functions [58].

### Independent verification of key proteomic findings by immunoblot analysis

Immunoblotting was used to confirm changed concentration levels of distinct muscle-associated proteins, as revealed by label-free LC–MS/MS analysis. Routine 1D gel electrophoresis and subsequent immunoblot analysis was performed by established methods [53]. The electrophoretic separation of WR and WT hind limb muscle samples was carried out with standard 10% (w/v) PAGE gels, followed by wet transfer at 100 V for 70 min to Whatman Protan nitrocellulose sheets in a Transblot Cell from BioRad Laboratories. To prevent non-specific antibody binding, membrane sheets were blocked for 1 h with a milk protein solution consisting of 5% (w/v) non-fat dried skimmed milk powder in phosphate-buffered saline. Incubation with sufficiently diluted primary antibodies to a variety of identified muscle proteins were carried out for a minimum of 3 h with gentle agitation at 4°C, followed by several washing steps and then incubation for 1 h at room temperature with sufficiently diluted peroxidase-conjugated secondary antibodies [55]. Following washing of membranes, the visualization of immuno-decorated protein bands was performed with the enhanced chemiluminescence method. The densitometric scanning and quantitative evaluation of immunoblots was carried out with an HP PSC-2355 scanner and GraphPad Prism and ImageJ (NIH) software.

## RESULTS

The MS-based proteomic analysis of 9-week-old WR hind limb muscle versus age-matched WT muscle was performed in order to establish the extent of altered protein concentrations in an established mouse model of motor neuron disease. Protein changes were determined by label-free LC–MS/MS analysis and alterations in proteomic hits of interest were confirmed by comparative immunblotting.

### Label-free LC–MS/MS analysis of WR versus WT hind limb muscle

The proteomic profiling of the WR muscle showed significant alterations in the abundance of 72 proteins species. As listed in Table 1, a decrease was determined for nine proteins and an increased concentration was found in the case of 63 proteins. Changes of 2-fold or higher were observed for 20 increased proteins and two decreased proteins. More moderate alterations in the expression levels occurred in 50 other muscle-associated proteins. Proteins that exhibited a considerable increase in the concentration were identified as a variety of contractile elements, including myosin light chains, troponin subunits TnI, TnC and TnT, and tropomyosin. In addition, the nuclear envelope protein lamin, the mitochondrial enzyme aldehyde dehydrogenase, histone protein H4, the molecular chaperone αB-crystallin, fatty acid-binding protein, fructose-1,6-bisphosphatase, four and a half LIM domains protein 1, carbonic anhydrase CA3, heat shock protein beta-1, annexin and peptidyl-prolyl *cis*–*trans* isomerase A were drastically increased in WR muscle. The haemoglobin subunits alpha and beta were both shown to be approximately 2-fold increased, suggesting that the WR and WT muscle fibres of hind limbs are possibly not equally well vascularized. The increased levels of haemoglobin may be due to higher levels of blood supply to WR muscles. However, since the animals had not been perfused prior to sacrificing, it is also possible that this result is an artefact of the isolation procedure and therefore both the WR and WT tissues may be equally well vascularized.

**Table 1:** List of proteins with a changed concentration in WR leg muscle as revealed by label-free mass spectrometry

Accession number	Protein name	Peptides	Score	ANOVA	Fold change
P05977	Myosin light-chain MLC1/3, muscle	2	126.35	0.0187	179.27
Q9WUZ5	Troponin I, slow	3	180.85	0.0047	7.91
P19123	Troponin C, slow	4	296.17	0.0016	6.25
O88346	Troponin T, slow	3	181.3	0.0050	6.09
P21107	Tropomyosin, alpha-3 chain	4	438.44	0.0020	4.69
Q91Z83	Myosin-7 (MyHC slow)	33	3080.77	0.0012	4.30
P51667	Myosin light-chain MLC2, cardiac	5	270.34	0.0086	3.90
P48678	Prelamin-A/C	9	592.98	0.0034	2.93
P47738	Aldehyde dehydrogenase, mitochondrial	4	236.63	0.0020	2.93
P09542	Myosin light-chain MLC3	4	365.98	0.0143	2.75
P62806	Histone H4	3	228.77	0.0010	2.59
P23927	αB-Crystallin (HSPB5)	3	170.51	0.0088	2.57
P04117	Fatty acid-binding protein, adipocyte	3	261.47	0.0007	2.48
P70695	Fructose-1,6-bisphosphatase isozyme 2	2	109.38	0.0013	2.34
P97447	Four and a half LIM domains protein 1	3	187.59	0.0030	2.34
P02088	Haemoglobin, subunit beta-1	5	480.94	0.0103	2.30
P16015	Carbonic anhydrase CA3	6	521.62	0.0152	2.23
P14602	Heat shock protein beta-1 (HSPB1, Hsp27)	3	153.41	0.0004	2.22
P07356	Annexin A2	3	159.34	0.0196	2.04
P17742	Peptidyl-prolyl *cis*–*trans* isomerase A	3	171.81	0.0231	1.99
Q8VHX6	Filamin-C	20	1399.68	0.0003	1.95
P60710	Actin, cytoplasmic 1	4	393.11	0.0327	1.89
P01942	Haemoglobin, subunit alpha	4	388.07	0.0192	1.89
P67778	Prohibitin	2	114.26	0.0210	1.83
P54071	Isocitrate dehydrogenase [NADP], mitochondrial	3	248.98	0.0024	1.72
P20152	Vimentin	6	347.23	0.0105	1.68
Q9JI91	Alpha-actinin-2	16	1373.39	0.0008	1.64
Q9D0K2	Succinyl-CoA:3-ketoacid coenzyme A transferase 1, mitochondrial	2	89.7	0.0041	1.63
Q9JKB3	DNA-binding protein A	2	167.62	0.0021	1.63
O08539	Myc box-dependent-interacting protein 1	4	215.92	0.0067	1.60
P07724	Serum albumin	11	792.98	0.0006	1.56
P20029	78 kDa glucose-regulated protein	2	225.91	0.0033	1.55
P62259	14-3-3 protein epsilon	3	188.9	0.0091	1.55
P31001	Desmin	9	705.13	0.0041	1.54
Q04857	Collagen, alpha-1(VI) chain	4	267.81	0.0146	1.52
P10126	Elongation factor 1-alpha 1	3	171.89	0.0294	1.50
Q02788	Collagen, alpha-2(VI) chain	5	317.3	0.0306	1.49
Q99JY0	Trifunctional enzyme subunit beta, mitochondrial	7	497.11	0.0060	1.49
P51885	Lumican	2	126.04	0.0145	1.49
P11499	Heat shock protein HSP 90-beta (HSPC)	5	375.59	0.0060	1.47
Q7TQ48	Sarcalumenin	6	381.25	0.0039	1.47
P51174	Long-chain specific acyl-CoA dehydrogenase, mitochondrial	3	165.65	0.0004	1.44
Q9JIF9	Myotilin	4	296.4	0.0063	1.43
Q61171	Peroxiredoxin-2	2	119.91	0.0228	1.40
P63038	60 kDa heat shock protein, mitochondrial	2	135.87	0.0034	1.39
P58252	Elongation factor 2	3	194.77	0.0121	1.39
Q8QZT1	Acetyl-CoA acetyltransferase, mitochondrial	2	134.75	0.0020	1.38
P68372	Tubulin beta-4B chain	2	548.87	0.0045	1.36
Q8BMS1	Trifunctional enzyme subunit alpha, mitochondrial	6	360.96	0.0166	1.36
Q99LC5	Electron transfer flavoprotein subunit alpha, mitochondrial	2	210.91	0.0181	1.35
P05202	Aspartate aminotransferase, mitochondrial	2	109.34	0.0083	1.32
Q91ZJ5	UTP-glucose-1-phosphate uridylyltransferase	2	117.55	0.0095	1.31
P56480	ATP synthase, subunit beta, mitochondrial	6	463.83	0.0263	1.31
Q9JKS4	LIM domain-binding protein 3	2	163.43	0.0020	1.29
P50544	Very long-chain specific acyl-CoA dehydrogenase, mitochondrial	4	261.63	0.0019	1.29
P38647	Stress-70 protein, mitochondrial	2	108.01	0.0009	1.27
Q9D2G2	Dihydrolipoyllysine-residue succinyltransferase component of 2-oxoglutarate dehydrogenase complex, mitochondrial	2	98.57	0.0163	1.26
Q99KI0	Aconitate hydratase, mitochondrial	5	304.47	0.0168	1.26
Q9DB20	ATP synthase subunit O, mitochondrial	2	117.63	0.0425	1.25
P63017	Heat shock cognate 71 kDa protein	4	407.65	0.0170	1.24
Q9DCW4	Electron transfer flavoprotein, subunit beta	3	201.29	0.0171	1.24
Q03265	ATP synthase subunit alpha, mitochondrial	4	365.7	0.0168	1.23
Q62234	Myomesin-1	3	176.76	0.0467	1.22
O88990	Alpha-actinin-3	4	431.15	0.0131	0.83
Q9WUB3	Glycogen phosphorylase, muscle	5	343.1	0.0130	0.79
A2ASS6	Titin	42	2579.98	0.0005	0.79
Q9D0F9	Phosphoglucomutase-1	6	438.29	0.0105	0.75
Q3V1D3	AMP deaminase 1	3	218.71	0.0271	0.73
Q8R429	Sarcoplasmic/endoplasmic reticulum calcium ATPase 1	5	398.2	0.0042	0.61
Q5SX39	Myosin-4, Mus musculus	4	990.28	0.0179	0.55
Q5XKE0	Myosin-binding protein C, fast-type	4	268.17	0.0291	0.51
P07759	Serine protease inhibitor A3K	2	154.17	0.0086	0.28

**Figure 1:**
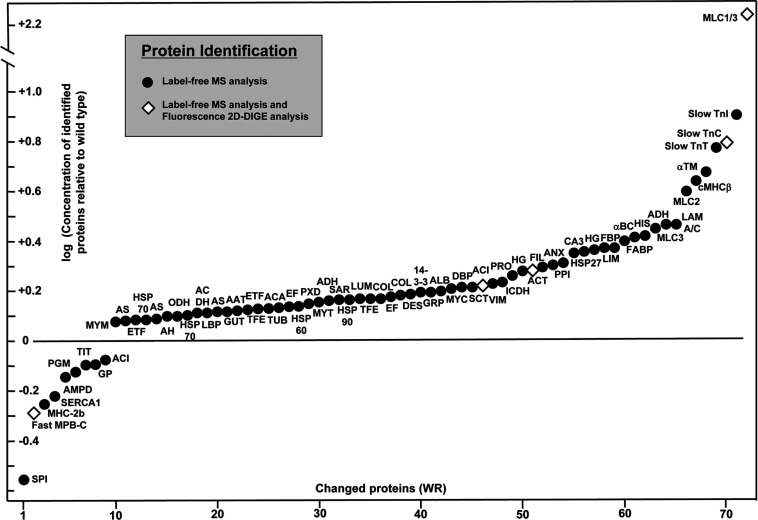
Overview of alterations in WR muscle proteins The graph shows the change in concentrations of muscle proteins identified by MS-based proteomics. Decreased versus increased proteins are shown in the lower and upper part of the graph, respectively. Proteins that have been identified by label-free MS analysis are marked by a solid circle and proteins which concentration changes have previously been established by fluorescence 2D in-gel electrophoretic analysis [39] are marked by a rhombus.

**Figure 2:**
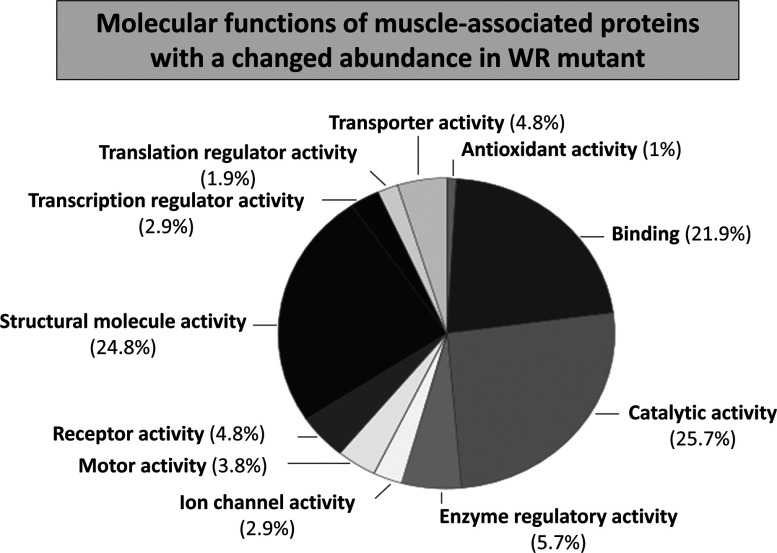
Molecular function of WR muscle proteins with a changed abundance Publically available bioinformatics software was used to identify the clustering of molecular functions of MS-identified muscle proteins with a changed abundance in the WR mouse model of ALS (Table 1). The analysis was performed with the PANTHER database (version 8.1) of protein families for the cataloguing of molecular functions [58].

An overview of protein expression changes in WR muscle is graphically presented in Figure 1. Muscle proteins associated with the contractile apparatus, metabolic pathways, metabolite transport, ion handling, structural maintenance and the cellular stress response are shown. Decreased proteins and increased proteins are shown in the lower and upper parts of the graph, respectively. Muscle proteins that have been identified by label-free MS analysis are symbolized by solid circles and proteins previously characterized by gel-based proteomics [39] are marked by a rhombus. While the previous application of fluorescence difference in-gel electrophoresis resulted in the identification of 14 changed proteins, with several proteins being recognized to be present in more than one protein spot [39], the label-free MS analysis determined altered expression levels in a considerably larger number of proteins. The fact that the previously identified increase in glyceraldehyde-3-phosphate dehydrogenase was not recognized by label-free MS analysis could be due the fact that the protein is better separated by 2D gel electrophoresis as compared with LC and/or that this glycolytic protein digests in an optimum way by in-gel methodology to produce large numbers of detectable peptides. Both proteomic techniques established similar findings for the fast isoform of MBP-C, actinin, actin, troponin and myosin light chain [39]. The application of the bioinformatics PANTHER database of protein families [58] revealed a list of molecular functions of the newly identified WR muscle proteins with an altered concentration. Figure 2 shows the cataloguing of the proposed functions in a pie chart: transporter activity (4.8%), antioxidant activity (1%), binding activity (21.9%), catalytic activity (25.7%), enzyme regulator activity (5.7%), ion channel activity (2.9%), motor activity (3.8%), receptor activity (4.8%), structural molecule activity (24.8%), transcription regulator activity (2.9%) and translation regulator activity (1.9%).

### Verification of proteomic findings by comparative immunoblotting of WR versus WT hind limb muscle preparations

**Figure 3:**
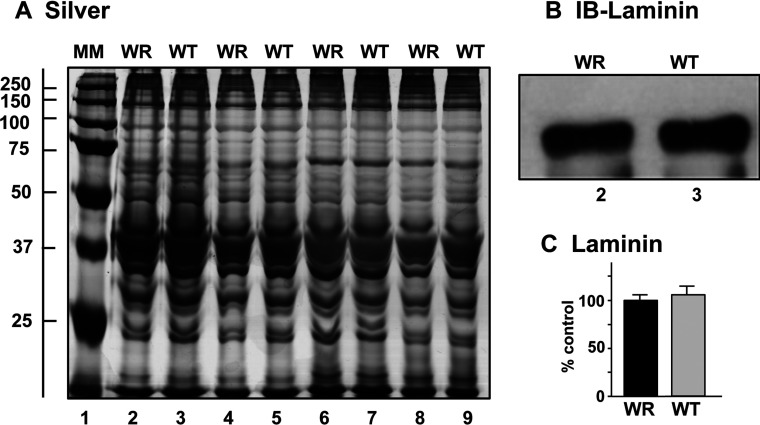
Electrophoretic analysis of WR and WT muscle preparations used for LC–MS/MS analysis Shown are a silver-stained 1D gel (**A**) and a representative immunoblot with an expanded view of immuno-decorated bands labelled with an antibody to laminin (**B**). Lane 1 shows MM (molecular mass) standards with their values in kDa on the left side of the panel (**A**). Lanes 2–9 represent four biological repeats of WR versus normal WT muscle preparations, respectively. The extracellular matrix protein laminin did not show significant changes in its concentration between WR and WT muscle extracts (**C**).

**Figure 4:**
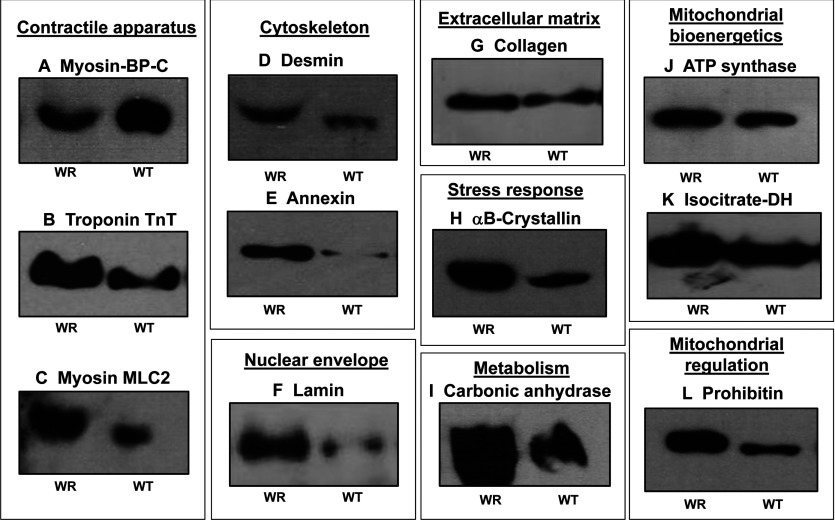
Immunoblot survey of proteins with a changed abundance in WR muscle as revealed by proteomics Shown are representative immunoblots with expanded views of immuno-decorated bands labelled with antibodies to MBP-C (**A**), troponin subunit TnT (**B**), myosin light chain MLC2 (**C**), desmin (**D**), annexin (**E**), lamin (**F**), collagen (**G**), αB-crystallin (HSPB5) (**H**), carbonic anhydrase isoform CA3 (**I**), mitochondrial ATP synthase (**J**), isocitrate dehydrogenase (**K**) and prohibitin (**L**). Lanes 1 and 2 represent WR versus WT muscle preparations, respectively.

**Figure 5:**
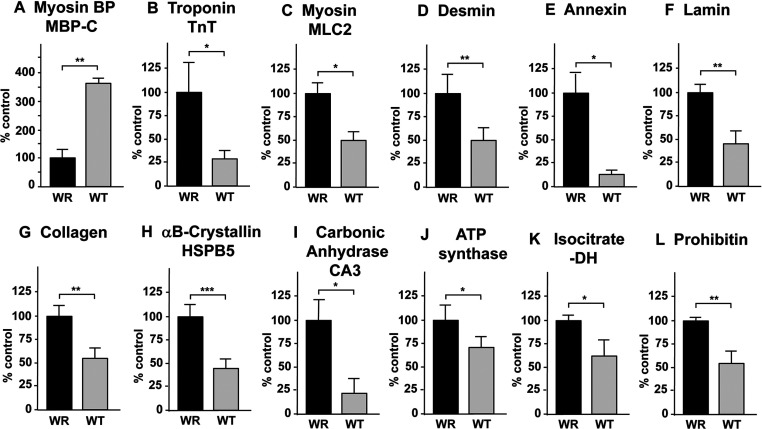
Densitometric analysis of the immunoblot survey of proteins with a changed abundance in WR muscle The comparative immunoblot analysis depicted in Figure 4 was statistically evaluated using an unpaired Student's *t* test (n=5; *P<0.05; **P<0.01; ***P<0.001).

**Figure 6:**
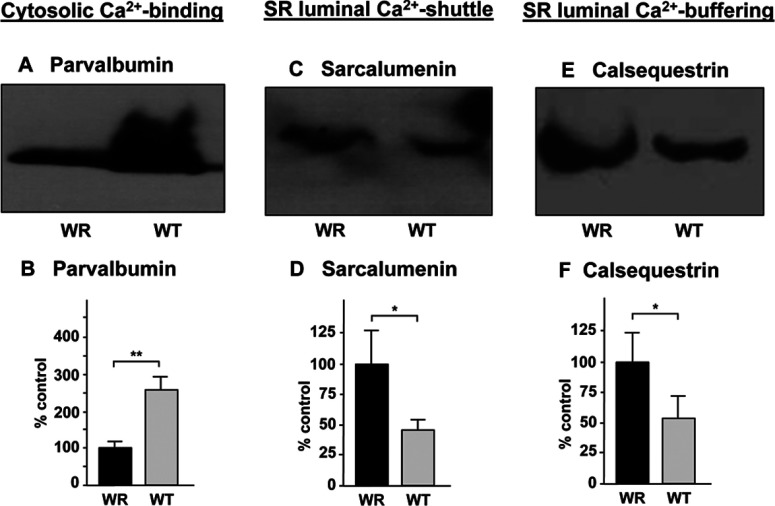
Immunoblot analysis of Ca^2+^-binding proteins with a changed abundance in WR muscle Shown are representative immunoblots with expanded views of immuno-decorated bands labelled with antibodies to the cytosolic Ca^2+^-binding protein parvalbumin (**A**, **B**), the luminal Ca^2+^-shuttle protein sarcalumenin of the SR (sarcoplasmic reticulum) (**C**, **D**) and the luminal Ca^2+^-binding protein calsequestrin of the terminal cisternae region of the SR (**E**, **F**). Lanes 1 and 2 represent WR versus WT muscle preparations, respectively. The comparative immunoblot analysis was statistically evaluated using an unpaired Student's *t* test (n=5; *P<0.05; **P<0.01).

Following the MS identification of protein changes in WR leg muscle, a variety of proteomic hits were verified by immunoblot analysis (Figures 3–6). This included marker proteins of the contractile apparatus, the cytoskeleton, the nuclear envelope, the extracellular matrix, the cellular stress response, muscle metabolism, mitochondrial bioenergetics and mitochondrial regulation. Figure 3 shows a silver-stained gel of four biological repeats of preparations from WR versus WT muscle, illustrating equal protein loading and relatively comparable protein band patterns in the two different muscle specimens. Immunoblotting with an antibody to laminin demonstrated comparable levels of this major extracellular matrix protein in atrophying and normal muscle samples (Figures 3B and 3C).

In contrast, significant concentration changes were confirmed for a range of muscle-associated proteins. While MBP-C was decreased in WR muscle (Figures 4A and 5A), the contractile proteins troponin TnT and MLC2 exhibited increased levels (Figures 4B, 4C; 5B, 5C). The nuclear envelope protein lamin, the cytoskeletal element desmin and the matrix–cell interaction protein annexin showed an increased concentration in WR muscle (Figures 4D–4F and 5D–5F). In contrast to the unchanged levels of the basal lamina component laminin (Figures 3B and 3C), collagen was found to be increased in WR muscle (Figures 4G and 5G). In agreement with cellular stress and modifications of the contractile apparatus, a drastically increased concentration of αB-crystallin was confirmed by antibody decoration (Figures 4H and 5H). In addition, the enzymes carbonic anhydrase CA3, mitochondrial ATP synthase and isocitrate dehydrogenase, as well as the regulatory protein prohibitin exhibited changes in their expression levels (Figures 4I–4L and 5I–5L) in agreement with the proteomic results listed in Table 1.

Figure 6 summarizes the immunoblot analysis of three crucial Ca^2+^-binding proteins, i.e. the cytosolic protein parvalbumin, the luminal Ca^2+^-shuttle protein sarcalumenin and the abundant Ca^2+^-buffering protein calsequestrin of the terminal cisternae region of the SR (sarcoplasmic reticulum). While parvalbumin was shown to be greatly reduced in WR muscle (Figures 6A and 6B), the luminal Ca^2+^-binding proteins exhibited increased levels in atrophying muscle preparations (Figures 6C–6F).

## DISCUSSION

Distinct changes in innervation patterns have a profound influence on skeletal muscle differentiation, fibre regeneration and the reprogramming of motor units during physiological adaptations to changed functional demands. The proteomic profiling of muscle plasticity has established discrete changes in the abundance and expression pattern of contractile proteins and metabolic enzymes during myogenesis and fibre maturation, as well as in response to enhanced neuromuscular activity, disuse-associated atrophy or chronic electrostimulation [18]. Here, we have used the WR mouse model of primary neuronopathy [43,51] to evaluate the complexity of proteome-wide changes in response to disease-induced alterations in fibre innervation. The label-free MS analysis of proteome-wide changes in neurogenic muscular atrophy has noticeably shown that distinct differences exist between the effects of nerve crush or complete denervation of skeletal muscles versus the influences of progressive degeneration of discrete motor neuron populations. The downstream biochemical effects of altered innervation patterns on neuromuscular modulation are clearly reflected by drastic changes in the concentration levels of muscle proteins. Importantly, this study has established that a more complex pattern of proteome-wide changes occurs in disease-associated muscular atrophy as compared with muscular disuse or denervation [18]. The proteomic analysis of WR muscle agrees with the idea that fibre-type specification and the metabolic weighting of bioenergetic pathways is clearly influenced by both a differing degree of a subtype-specific vulnerability of neuromuscular synapses and compensatory mechanisms of fibre-type shifting in motor neuron disease. In general, experimental denervation, traumatic nerve injury or prolonged neuromuscular disuse results in relatively unilateral skeletal muscle transitions. While enhanced neuromuscular activity triggers a clear fast-to-slow transition in skeletal muscle, disuse-related muscular atrophy or complete denervation is mostly associated with a slow-to-fast transformation. In contrast, the new proteomic data presented here demonstrate that protein changes in motor neuron disease are more complex and that this disease is not associated with a straightforward slow-to-fast transformation process. Motor neuron disease appears to be associated with complex differential expression patterns of both fast and slow muscle protein isoforms.

In agreement with the findings of a previous DIGE-based analysis of WR muscle [39], disease-associated muscular atrophy causes a reduced concentration of the fast MBP-C. Immunoblot analysis has confirmed the lower concentration of this contractile protein in WR muscle. In addition, the study presented here has established a reduction in α-actinin, titin and myosin 4, the foetal muscle isoform of MHCIIb, indicating remodelling of the cytoskeletal network and the auxiliary structure and contractile elements of the actomyosin apparatus [59]. The lower concentration of glycogen phosphorylase and phosphoglucomutase agrees with the idea of a disturbed glucose metabolism in WR muscle. However, the glycolytic enzyme fructose-1,6-bisphosphatase was found to be increased in WR preparations, which was also shown in a previous gel-based study of WR muscle [39]. Since some glycolytic enzymes are multi-functional and are also potentially involved in cellular signalling [60], the differential expression levels of individual glycolysis-related proteins may reflect changes in other biochemical processes than anaerobic metabolism [39,40]. The complex alterations in glycolytic and mitochondrial muscle enzymes shown in this study indicate that the metabolic transitions in WR muscle is more complex than a unidirectional shift towards a particular bioenergetic phenotype.

Interestingly, the SERCA1 (sarcoplasmic/endoplasmic reticulum Ca^2+^-ATPase 1) isoform of the relaxation-inducing Ca^2+^-ATPase of the SR was identified by label-free MS analysis. This finding illustrates suitably the advantages and complementary nature of gel-free separation approaches in muscle proteomics and shows that liquid chromatography-coupled MS analysis is capable of detecting changes in integral and highly hydrophobic muscle enzymes [61]. Since the Ca^2+^-pumping ATPase is a relatively abundant protein, it is difficult to interpret whether changes in the SERCA1 isoform play a major role in the atrophy-related fibre changes or represent a minor alteration within the extensive network of the SR membrane system. Immunoblotting with antibodies to the Ca^2+^-ATPase from the SR resulted in excessive background staining (not shown), which prevented a proper verification of this proteomic finding. Interestingly, the concentration of the luminal Ca^2+^-binding proteins sarcalumenin and calsequestrin is considerably increased in WR muscle, which is indicative of a greater demand for Ca^2+^-buffering within the SR. A possible reason for this is the avoidance of Ca^2+^-dependent proteolysis and/or abnormal cellular signalling on the level of excitation–contraction coupling, as is commonly observed in muscular disorders [62]. As previously shown by immunoblotting and proteomics in the WR and SOD mouse models of ALS [39,40], respectively, the concentration of the cytosolic Ca^2+^-binding protein parvalbumin is drastically lowered in motor neuron disease. This reduced capability of cytosolic Ca^2+^-buffering might be related to the increased levels of luminal Ca^2+^-binding capacity in WR muscle preparations and agrees with previously described decreased mRNA levels of parvalbumin in the same animal model of ALS [63].

In contrast to the above outlined decreases in metabolic enzymes and contractile proteins, a large number of proteins were found to be increased in WR muscle. This included proteins involved in muscle contraction, the cytoskeleton, the extracellular matrix, metabolite transportation, muscle metabolism, mitochondrial regulation, ion handling and the cellular stress response. The immunoblot survey presented in this study has confirmed these concentration changes in a variety of marker proteins of distinct subcellular regions of skeletal muscle tissue. Striking increases were observed for several slow isoforms of myosin light chains and the TnI, TnC and TnT subunits of the troponin complex [59]. Thus, in divergence to unilateral slow-to-fast transitions due to chronic unloading or experimental denervation procedures, as shown by various proteomics studies [64–68], the increase in slow forms of essential contractile proteins probably reflects an initial preferential loss of the fast type of neuromuscular synapses in the WR model of motor neuron disease. Increased levels of a variety of mitochondrial components such as aldehyde dehydrogenase, isocitrate dehydrogenase, prohibitin, succinyl-CoA:3-ketoacid coenzyme A transferase, long-chain specific acyl-CoA dehydrogenase, trifunctional enzyme, electron transfer flavoprotein, aspartate aminotransferase, acetyl-CoA acetyltransferase, aconitate hydratase, oxoglutarate dehydrogenase and ATP synthase would agree with this idea. These alterations are therefore probably not directly related to an organized or adaptive pattern of fibre-type shifting, but more likely based on the initial consequence of a preferential loss of neuromuscular synapses that normally function within a fast type of innervation process.

The raised levels of the nuclear envelope protein lamin-A/C and the histone H4 protein indicate changes or restructuring of muscle nuclei within select fibre populations of the WR mouse. Lamin A/C and lamin B of the nuclear envelope form stabilizing LINC complexes with SUN1/2 proteins and nesprins, which in turn bind to cytoskeletal filaments [69]. Therefore an indirect link might exist with respect to other changed muscle proteins, such as filamin, desmin, tubulin and vimentin. Muscle-specific filamin C acts as an actin cross-linking protein, desmin and vimentin are intermediate filament components and tubulin constitutes an essential protein of microtubules [70]. Their increased abundance, as already previously reported [39], suggests a reorganization of the muscle cytoskeleton and interior filament network in response to neurodegeneration-related unloading. The extracellular matrix protein collagen and the multi-functional protein annexin A2, which binds to sarcolemmal dysferlin and is probably involved in cell–matrix interactions, were also shown to be increased, which could be a compensatory response to stabilize the weakened fibre periphery during disease-related fibre unloading. Since the surface membrane-associated dystrophin–glycoprotein complex and the neuromuscular junction-coupled utrophin–glycoprotein complex are closely linked to the actin membrane cytoskeleton [71], which is indirectly associated with the complex cytoskeletal filaments within the muscle interior and the outside structural support of the nuclear envelope, it is possible that external stimuli affect the entire cytoskeletal network of muscle fibres simultaneously, including the lamin-associated LINC complex [69]. Thus, motor neuron disease appears to be associated with considerable changes in the extracellular matrix, the actin cytoskeleton, intermediate filaments, microtubules and the nuclear envelope.

Elevated levels of the molecular chaperones αB-crystallin (HSPB5), Hsp27 (HSPB1, beta-1), the large heat shock protein HSP90-beta (HSPC), the stress-70 protein, the mitochondrial 78 kDa glucose-regulated protein, the mitochondrial 60 kDa heat shock protein and the heat shock cognate 71 kDa protein are indicative of considerable cellular stress levels in WR muscle and the need for extensive refolding or the swift removal of misfolded muscle proteins [72]. Increases in a variety of heat shock proteins were also reported by Capitanio et al. [40] to occur in the SOD mouse model of ALS. Thus, the missense mutation in the *Vps54* gene that causes a L967Q replacement in the vesicular protein-sorting factor VPS54 [51] triggers substantial cellular stress in affected muscle tissues. The destabilization of the tertiary protein structure of VPS54 and subsequent reduction in its abundance levels seems to severely disturb the function of the GARP complex and indirectly trigger an extensive stress response. The predominant initial effect on fast type synapses is substantiated by the fact that increased amounts of the CA3 isoform of carbonic anhydrase were identified in WR muscle. Since immunocytochemical studies by Fremont et al. [73] have demonstrated higher levels of carbonic anhydrase in *soleus* muscle as compared with *vastus lateralis* muscle, possibly changes in muscle mass and contractile function are focused initially on fast-twitching fibres populations in motor neuron disease. Alternatively, the pathophysiological changes in WR muscle may require an increased demand for efficient CO_2_-removal during fibre remodelling.

Recently, early gene expression changes were studied by microarray screening of gastrocnemius muscle from the SOD1 mouse model of ALS [74]. The investigation identified differential gene activation levels in the Wnt/PI3K (phosphoinositide 3-kinase) signalling pathways and epithelial–mesenchymal transitions in pre-symptomatic skeletal muscle. Since the PI3K pathway is involved both in the inhibition of cell death and the promotion of cell proliferation, the maintenance and repair of affected fibre populations may be impaired. Glucose uptake and fiber differentiation may also be altered, agreeing with the general idea of motor neuron disease being a multisystem disorder with a defective muscle metabolism. The gene expression changes observed in the *gastrocnemius* muscle of SOD1-G93A transgenic mice suggest that neuromuscular impairments precede motor neuron death at pre-symptomatic periods of motor neuron disease [74]. The analysis of muscle biopsies from ALS patients identified interesting alterations in the gene expression patterns in this form of motor neuron disease, including elevated levels of mRNA encoding myosin-8, collagen, actin and annexin [75]. Decreased mRNA levels of actinin alpha-3 and the fast isoform of MPB-C agree with the findings from this proteomic study. Since decreased levels of fast MBP-C were shown to occur in degenerating muscle by both label-free MS analysis and gel-based proteomics [39], as well as transcriptomic analysis of ALS muscle biopsies [75], this protein represents an excellent new candidate of an internal muscle-associated biomarker of motor neuron disease. Other transcriptomic analyses have focused on the spinal cord from animal models and ALS patients [76–78]. Altered patterns of cell adhesion, the immune response, lipid metabolism and inflammation were shown to be involved in the onset of progressive paralysis, muscular atrophy and a hypermetabolic state in motor neuron disease [78].

In conclusion, certain pathophysiological mechanisms involve the preferential loss of motor neurons and/or are associated with defective re-innervation patterns, such as muscle ageing or motor neuron diseases. Although primary motor neuronopathies evidently lead to muscular atrophy, the effect of progressive neurodegeneration on the contractile phenotype or bioenergetics processes is not as one sided as in the case of complete denervation. The preferential loss of certain types of motor neurons exerts a differential effect on fibre-type specification and metabolic pathways. The label-free MS analysis presented here could show that, in contrast to the characteristic slow-to-fast transitions associated with disuse-related muscular atrophy or denervation-associated fibre wasting, motor neuron disease exhibits more complex protein alterations on the level of metabolic enzymes and contractile proteins. There certainly exists a distinct link between changed neuromuscular functions and complex proteome-wide alterations in skeletal muscle. The newly identified alterations in muscle proteins can now be exploited as novel diagnostic, prognostic or therapeutic targets to improve the evaluation and treatment of motor neuron disease. Especially striking is the drastic reduction in the fast isoform of MBP-C [39,75], making this protein isoform a good candidate to be exploited as a new marker for designing improved assay systems.
